# Reconstruction of a full thickness nasal alar defect following resection of a BCC with a vascularised chondrocutaneous helical rim flap: a case report

**DOI:** 10.1186/1753-6561-9-S1-A29

**Published:** 2015-01-14

**Authors:** F Shiel, A Pabari

**Affiliations:** 1 Royal College of Surgeons in Ireland, 123 St. Stephen’s Green, Dublin 2, Ireland; 2 Department of Plastic and Reconstructive Surgery, The Royal Free, Hampstead NHS-Trust, London, UK

## Background

Nasal reconstruction presents a challenge for plastic surgeons due to the prominent location and complex structure of the nose. When reconstructing full-thickness nasal defects, options to replace the inner lining, cartilaginous framework and outer skin must be considered. The aesthetic units of the nose must also be replaced in full rather than simply filling holes. This report presents a case of a 62 year old male who’s full-thickness alar defect due to resection of a basal cell carcinoma (BCC) was reconstructed using a free chondro-cutaneous helical rim flap based on a retrograde flow superficial temporal artery pedicle.

## Methods

The margins of the existing defect were excised by a further 5mm as suggested by histology. A mould of the defect was made using bone cement and used to plan the harvest site of the flap from the left helical rim. The flap was raised based on a retrograde superificial temporal artery and consisted of cartilage from the helical rim, skin from the anterior and posterior surfaces of the helix and a small segment of post-auricular skin. A 10cm inter-positional pedicle was harvested from the descending lateral circumflex femoral artery and vein from the right antero-lateral thigh (ALT). This inter-positional ALT pedicle was tunnelled under the cheek to reach the facial artery and vein in the submandibular region as the vessels in the naso-labial fold were not suitable for anastomosis.

## Results

The vascularised free chondrocutaneous flap allows reconstruction of the three layers of the nasal ala: the inner lining, cartilaginous framework and outer skin, in one procedure. Due to similar sun exposure, the auricular skin also provides a good match to nasal skin in terms of colour and texture. The natural curvature of the helical rim at the root is similar to that of the nasal ala. The resulting ear defect with be reconstructed at a later stage using a cartilaginous graft from the costal margin and skin grafting.

## Conclusions

As 30% of head and neck BCC’s are nasal, consideration of reconstructive options following resection is an important part of patient management. The vascularised helical rim flap allows reconstruction of all 3 layers of the nasal ala with a good aesthetic result due to adequate matching of contour, colour and texture. The location of any scarring is inconspicuous in comparison to a local forehead flap that could also be used to reconstruct such a defect.

